# Excessive Gestational Weight Gain: Long-Term Consequences for the Child

**DOI:** 10.3390/jcm9123795

**Published:** 2020-11-24

**Authors:** Joanna Baran, Aneta Weres, Ewelina Czenczek-Lewandowska, Justyna Leszczak, Katarzyna Kalandyk-Osinko, Edyta Łuszczki, Grzegorz Sobek, Artur Mazur

**Affiliations:** 1 Institute of Health Sciences, Medical College, University of Rzeszów, Al. mjr.W.Kopisto 2 a, 35-310 Rzeszów, Poland; anetaweres.ur@gmail.com (A.W.); e.czenczek@univ.rzeszow.pl (E.C.-L.); leszczakjustyna.ur@gmail.com (J.L.); eluszczki@ur.edu.pl (E.Ł.); g.sobek@wp.pl (G.S.); 2 Institute of Medical Sciences, Medical College, University of Rzeszów, Al. mjr.W.Kopisto 2 a, 35-310 Rzeszów, Poland; kalandyk@op.pl (K.K.-O.); drmazur@poczta.onet.pl (A.M.)

**Keywords:** gestational weight gain, health, maternal, obesity, perinatal risk factors, pregnancy

## Abstract

Background: The aim of the study was to analyse the impact of mothers’ gestational weight gain (GWG) and age at birth on the long-term risk of overweight and obesity in preschool and school-aged children. Methods: The study involved 749 mothers and children at ages between four and 15 years old. Each child was assessed for height and body weight, and then, the body mass category was determined based on the body mass index (BMI) percentile according to the sex and age of the subjects. Information on the perinatal risk factors for overweight and obesity came from the child’s health card or mother’s maternity card. They contained information about the mother’s age at the time of childbirth and the mother’s gestational weight gain during pregnancy. Results: In the group of 7–11-year-olds, the maternal weight gain during pregnancy was higher in obese children than in children with normal weight (18.8 kg vs. 14.3 kg; *p* = 0.002). This relationship was shown analogously in the group of 7–11-years-olds boys (20.6 kg vs. 15.1 kg; *p* = 0.005). Positive correlations were also shown between mother’s gestational weight gain and the BMI percentage of the whole group (*p* = 0.004). In the case of the mother’s age, no statistically significant relationship was found with the child’s weight category. Conclusions: Mothers’ weight gain during pregnancy is a factor that promotes overweightness and obesity in the child. Maternal age at birth does not appear to lead to any propensity toward overweightness and obesity in the later life of a child.

## 1. Introduction

Obesity develops as a result of a prolonged supply of energy in excessive amounts in relation to its optimal expenditure and is increasingly observed in the paediatric population. The incidence of obesity among infants and young children up to five years old around the world has increased rapidly from 32 million in 1990 to 42 million in 2013, and this number is predicted to grow up to 70 million by 2025 [1,2]. Around 55% of obese children go on to be obese in adolescence, around 80% of obese adolescents will still be obese in adulthood and around 70% will be obese over age 30 [3]. For this reason, preventive activities, as well as the search for risk factors, should start already in early childhood. 

Although genetic factors play a very large role in the development of childhood obesity, this problem has significantly grown in highly developed countries, which indicates that environmental factors are equally important. Early risk factors are particularly noteworthy, including maternal ones, starting from the period of pregnancy and delivery [4,5].

Previous scientific reports confirm that a pregnant mother’s unhealthy lifestyle, including a poorly balanced diet [6,7], low physical activity [8] and smoking [9,10,11], may contribute to the abnormal birth weight of a child and excessive body mass index (BMI) in the subsequent years of life. This dependence has been confirmed in both preschool and school-aged children. In addition, it was estimated that the risk of overweightness and obesity increases up to twice as high in children whose mothers were obese before and during pregnancy [8,12,13].

According to the recommendations of the American College of Obstetricians and Gynecologists (ACOG), women who are underweight before pregnancy (BMI < 18.5 kg/m^2^) should increase their body weight in pregnancy by 12.7 to 18.1 kg, women with normal body masses (BMI between 18.5–24.9 kg/m^2^) by 11.3–15.9 kg and women who are overweight (BMI between 25.0–29.9 kg/m^2^) by 6.8–11.3 kg. Women in all classes of the obesity category (BMI > 30.0 kg/m^2^) should not gain more weight during pregnancy than 5–9.1 kg [14]. Therefore, the special care and education of future mothers is necessary in the field of healthy and rational nutrition in pregnancy and regarding the risk of excessive weight gain during this period. Maintaining a correct body weight before pregnancy and gestational weight gain (GWG) during pregnancy is important for both the health of the child and the mother herself. According to the literature data, the number of pregnant women who are overweight or obese before pregnancy is growing dramatically [15,16].

Nowadays, the next important issue is the mother’s age at birth, which, in recently measured years, has increased, particularly in high-income countries. Presently, it is known that an advanced maternal age (>35 years) may be associated with a higher risk of complications during childbirth, as well as with a greater predisposition of the child to many health disorders in the perinatal, paediatric and adulthood stages, including diabetes [17,18], hypertension [19], Alzheimer’s disease [20] and oncological diseases [21]. The association of this factor with childhood obesity is still under discussion and worth investigating more closely [22,23].

The aim of the study was to analyse the impact of mothers’ gestational weight gain and age at birth on the long-term risk of overweightness and obesity in preschool and school-aged children.

## 2. Experimental Section

### 2.1. Participants

In the in South-Eastern Poland where the research was carried out, in 2017, according to demographic data, 2,129,138 residents lived, and 51% of them were female and 49% were male. Girls accounted for 14.4% of the female population, and 15.8% of the male population were boys, of preschool and school ages [24]. Due to the fact that the occurrence of excessive body weight in children shows a large discrepancy with regard to sex, the sample size was calculated separately for girls and boys. The size of the required sample was calculated, taking into account a 95% confidence level and the estimated average occurrence of the phenomenon of excessive body weight at the level of 20% in girls and 26% in boys [25]. The level of significance was considered *p* < 0.05. It was calculated that the minimum sample size should be: 246 girls and 296 boys.

**Figure 1:**
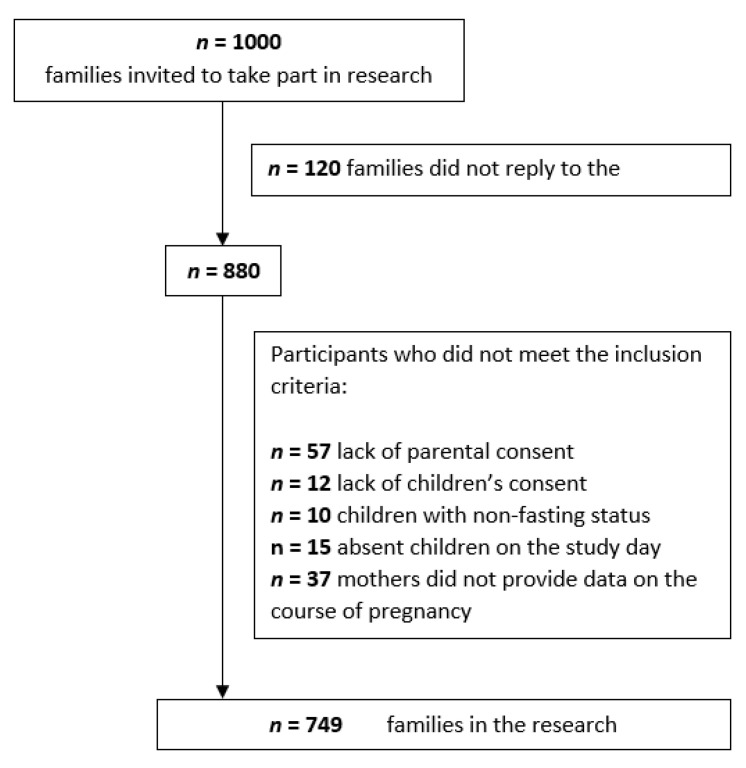
Flow chart of the participants.

Finally, the study involved 749 mothers and children. The detailed procedure for determining the participants is presented in Figure 1. After consent was obtained, it turned out that the age range of the subjects was within the range of 4–15 years. The children attended kindergartens, primary and junior high schools. The mean age of the examined children was 9.36 years ± 3.52 years.

The parents were informed about what the examination would involve and the possibility of withdrawing from participation at any time. Each participant underwent testing on an empty stomach, and further measurements were made during the morning hours. Each child’s height and weight were measured. In addition, the parents completed a questionnaire containing basic information about the child and their family and provided a photocopy of the child’s health book and mother’s pregnancy card.

### 2.2. Physical Examination

Body height measurement was performed using a Seca 213 stadiometer (Seca, Hamburg, Germany). Body height was measured three times, and the average value was taken in order to eliminate the measurement error. The device complies with Annex VI to the Directive 93/42/EEC concerning medical devices.

Body weight was assessed using the Tanita BC 420 MA analyser (Tanita, Tokyo, Japan), which meets all requirements in accordance with the European NAWI (Non-automatic weighing instruments) and MDD (Medical devices directive) standards and directives [26].

The subject stood with bare feet on the analyser, the upper limbs were placed along and slightly away from the torso and eyes directed straight ahead. The person performing the test entered the age and body height of the subject into the analyser software (Health Monitor GMON version 3.4.1, Tanita Japan Pro, Tokyo, Japan). After about 10 s, a result was obtained in the form of a printout.

The measurements were taken in the morning, by the same researcher, with the subject in a fasted state.

### 2.3. Maternal Factors

Information on the perinatal risk factors for overweightness and obesity came from the child’s health book and the mother’s pregnancy card. They contained information about the mother’s age at the time of childbirth and the weight gain of the mother during pregnancy.

After collecting all of the above data, each child’s current BMI percentile was determined with reference to the national percentile grids [27], and the child’s body mass category was determined on the basis of the BMI percentile, with reference to the classification by Barlow et al. [28].

### 2.4. Statistical Analysis

Data analysis was carried out using selected methods of descriptive statistics and statistical inference. Selected numerical characteristics of the tested parameters were determined: number (*n*), percent (%), mean (x), median (Me) and standard deviation (*s*). 

To determine the level of statistical significance, the Mann-Whitney nonparametric test was used. The statistical significance level was *p* < 0.05. 

The Spearman rank correlation coefficient was applied to analyse the relationship between the two measurable features. The results were supplemented with the results of the significance test of the correlation coefficient (*p*), which allowed the assessment of whether the dependence found in the sample reflected a more general relationship prevailing in the entire population or just a matter of chance.

A multifactor analysis of variance (ANOVA) was also used. Using the analysis of variance, the significance of differences between groups was determined by individual variables (the so-called main effects), and the effects of interactions between the factors were tested. Calculations were performed with Statistica 10.0 (StatSoft, Poland).

### 2.5. Ethical Approval

The study was approved by the local Bioethics Committee No. 18/12/2015 of 2 December 2015.

## 3. Results

The women were an average of 27.2 years ± 4.8 years old during their pregnancy and gained an average of 15.1 ± 6.5 kg. The range of gestational weight gain was between 3 and 35 kg.

It was also shown that during the pregnancy that the mothers of boys grew 1.3 kg more than the mothers of girls (*p* = 0.021). The age of the mother at the time of delivery did not differ significantly in the cases of girls and boys (Table 1).

The mother’s age during pregnancy did not have a clear impact on the current body weight of the children in the study group. In the group of girls, older mothers more often gave birth to children who currently have a normal body weight. Among boys, the situation was reversed. These results are not statistically significant; therefore, they cannot be the basis for statistical inference.

The analysis showed that mothers of children who are currently overweight or obese gained more weight during pregnancy than mothers of children with normal body weight. This was especially noticeable among boys (*p* = 0.005) and the whole group of children aged 7–11 (*p* = 0.002) (Table 2).

The effect of the mother’s gestational weight gain on the BMI percentile classification was statistically significant in the group of 7–11-year-olds (*p* < 0.001) and at the level of the whole population without an age distribution (*p* = 0.004). In the remaining age groups, no statistically significant relationships were found.

Analysing the correlations with regards to the sex of the child, statistically significant positive correlations were shown between the mother’s gestational weight gain and the BMI percentage of all girls (*p* = 0.017), 7–11-year-old girls (*p* = 0.017) and 7–11-year-old boys (*p* = 0.014) (Table 3).

The results of a comprehensive survey of the relationship between gender, age and BMI classification with maternal perinatal factors using a three-factor analysis of variance is presented in Table 4.

These results confirmed the relationship between body mass index by BMI percentile and the absolute increase in maternal body weight during pregnancy. The nature of this dependence depended on age, which resulted from the significance of the statistical interaction of the age and BMI classification.

**Figure 2:**
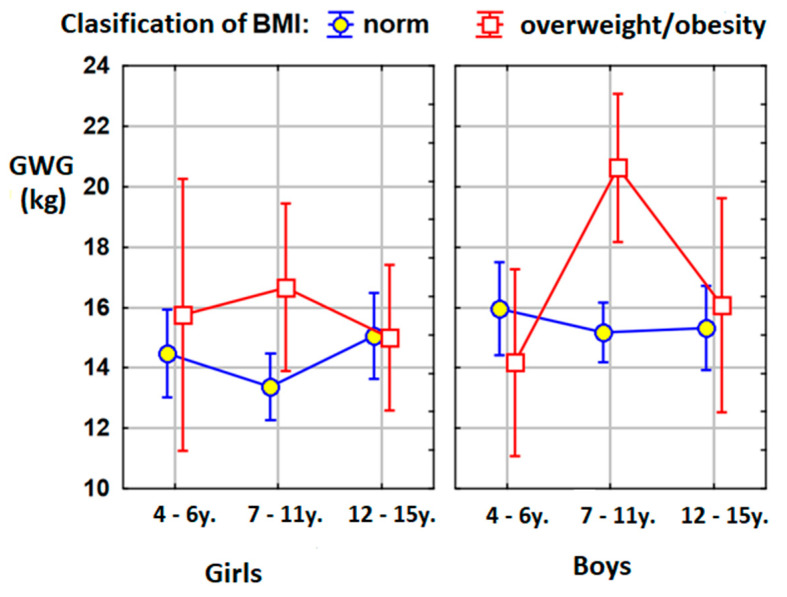
Graphic presentations of the means in the compared groups. BMI: body mass index; kg: kilograms; GWG: gestational weight gain.

The confirmation of these results is shown in Figure 2 below.

## 4. Discussion

In the modern day, childhood obesity is described as a chronic disease with a multifactorial aetiology, which is influenced by both genetic and environmental factors. Maternal body weight before pregnancy and gestational weight gain (GWG) are considered the two most important environmental determinants that influence the early development of childhood obesity. It has also been proven that the method and type of maternal nutrition during pregnancy may program the expression of some genes in the embryo, which may be responsible in the future for the appearance of some metabolic disorders, such as insulin resistance, hyperlipidaemia, hypertension and abdominal obesity. Excessive GWG causes permanent changes in the metabolism similar to those in the case of gestational diabetes mellitus (GDM) [29,30].

One of the analysed factors that may play a role in the development of overweightness and obesity in children’s futures was the mother’s age at delivery. As shown by the results of our own work, no significant relationship was found. Although the available literature often deals with the subject of various characteristics of the mother and their importance for the broadly understood health of the child at the time of delivery and in the future, it is rarely mentioned by the authors in the context of overweightness and obesity.

The studies carried out did not show any relationship between the mother’s age during pregnancy and the increased occurrence of overweightness and obesity in the school-age children. In the group of girls, younger mothers more often gave birth to children who are currently overweight or obese. The difference was between 0.1 to 1.2 years of age, depending on the current age group of the children. Among the boys, the situation was reversed. Older mothers gave birth to more children who are currently overweight or obese. The difference was between 0.2 to 2.6 years of age, depending on the current age group of the boys. These results were not statistically significant; therefore, they cannot be the basis for statistical inference.

The literature provides data on this subject; however, scientists’ opinions are divided as to whether maternal age affects the risk of obesity in children or not. The correlation of maternal factors in the perinatal period with the development of overweightness and obesity among children (aged four–six) was investigated by, among others, Varela-Silva et al. The authors reached similar conclusions as in our own study, as they stated that maternal age is not a predictor of overweight children. The authors attributed a greater role to maternal body height and child’s birth weight. Children with a mother shorter than 150 cm were less than half as likely to be overweight, and children with birth weights below 3000 g were only a third as likely to be overweight as their peers within the range of normal birth weight (3000–3500 g) [31].

The results of our research are also consistent with the data presented by Mourtakos et al. [32]. They reported that the mother’s age during pregnancy was not a factor increasing the risk of overweightness and obesity in the child. 

Woo Baidal et al., carrying out a broad analysis of the risk factors for obesity in children during the first 1000 days of life, pointed out that almost 300 prospective studies confirmed the significant role of maternal factors: in particular, excessive BMI of the mother before pregnancy, excessive pregnancy weight and exposure to tobacco smoke. To date, there is no conclusive evidence that the mother’s age at birth would have an adverse effect on the weight gain of the child, and our own study did not confirm this assumption [33]. 

Similar conclusions were obtained from the research by Janjua et al. They showed that factors such as the mother’s employment status, mother’s age, father’s education, race/ethnicity, quality of home environment and the number of adults in the home were not significantly related to childhood obesity [34].

The authors of other works presented a different view, suggesting that maternal age has an impact in relation to offspring health and health behaviours in late adolescence. Research suggests that the mother’s age at the time of delivery is of great importance for the future development and functioning of the baby, as people who were born to older mothers have lower self-rated health, were more likely to smoke and drink alcohol regularly and less likely to exercise regularly, which can lead to obesity [35].

Other studies found that the predicted risk of overweightness and obesity in childhood decreased with increasing the maternal age. The results found that caesarean delivery and maternal age <28 years might be predictors for overweightness and obesity, the interaction of which increased at least twofold the risk of overweightness and obesity compared with others, especially in boys aged over five years. [36]. These results are opposite to the results of our own research, where boys born to older mothers were prone to obesity today. According to Saunders, the maternal age is associated with paediatric overweightness and obesity [37]. This statement is in opposition to the results of our own research. Although little research is available on the relationship between maternal age and paediatric obesity, the results of previous studies were inconsistent and inconclusive.

The second factor we analysed, which has a potential impact on the occurrence of overweightness and obesity in children, was the increase in the mother’s weight during pregnancy. Despite the publication of the Institute of Medicine’s (IOM) recommendations in 2009, many women attain GWG above these recommendations, especially in the third trimester. Even the majority of women with normal BMI before pregnancy experienced GWG above recommendations, with overweight and obese women particularly at risk. Although exceeding the GWG compared to the recommendations is the most common phenomenon, there are also cases involving GWG below the recommendations, especially among women who are underweight [38]. A higher than average GWG accounts for a moderate increase in the risk of the offspring being overweight, whereas a lower than average GWG does not appear to reduce this risk [39].

Our study confirmed significant relationships between the mother’s weight during pregnancy and the child’s current body mass category. This was confirmed by the meta-analysis of Tie et al. [40] and studies by Lau et al. [41] and, also, Leonard et al., where an excessive GWG was associated with an increased risk of a high birthweight and overweightness in 2–5-year-olds, 6–11-year-olds and 12–19-year-olds in the total population [42]. 

Many other authors described similar observations. Excessive weight gain during pregnancy clearly increased the risk of excessive body weight in children, starting from an early age, through preschool and school ages [43,44,45]. A logistic regression analysis by Shao et al. showed that pre-pregnancy overweightness and obesity (odds ratio (OR) = 2.01, 95% confidence interval (CI): 1.53–2.65) and excessive GWG (OR = 1.65, 95% CI: 1.35–2.03) were risk factors for overweightness and obesity, and pre-pregnancy underweightness was a protective factor for childhood overweightness and obesity (OR = 0.49, 95% CI: 0.39–0.62) [46]. The analysis performed by Leonardo et al. showed that the risk of obesity among children was the lowest in the group with the mothers of the lowest body weights. Differences in the risk of obesity were the highest after the age of five and persisted during adolescence. In addition, if the mother was already overweight pre-pregnancy, this was associated with a more than twofold increase in the risk of obesity in the children aged 6–11 (adjusted relative ratio (RR): 2.39, 95% CI: 1.97–2.89) and 12–19 years (adjusted RR: 2.74, 95% CI: 2.13–3.52) [47].

Numerous studies also indicated that mothers with GWG higher than the recommendations tend to give birth to larger children. In the future, these children are more likely to be exposed to higher BMI and the occurrence of cardiometabolic diseases, including obesity. This applies to both children in the first years of life, as well as older children and those from different regions of the world characterised by different diets. In spite of other complicating factors, excessive GWG of the mother is a decisive factor in the appearance of excessive body weight in the child [32,48,49,50].

Another study showed that an increase in GWG by 1 kg was associated with an increase of 0.009 (95% confidence interval (CI): 0.007–0.010, *p* < 0.001) in the mean BMI in children; in the subgroup of mothers who were overweight/obese before pregnancy, the increase in BMI in the children was 0.028 (95% CI, 0.017–0.039, *p* < 0.001). Excessive weight gain during pregnancy played an important role in overweightness in children when the GWG was used as a benchmark with an odds ratio (OR) of 1.21 (95% CI, 1.12–1.29). The risk was highest (OR 2.22, 95% CI, 1.79–2.76) in children of mothers who were overweight/obese before pregnancy and who gained excessive weight during pregnancy. In conclusion, a greater GWG in the mother was associated with a higher BMI in the offspring, and the risk of being overweight doubled in children whose mothers were overweight/obese before pregnancy and who gained excessive weight during pregnancy [12].

Similar results were presented by Wan et al., who found a BMI score for children on average 0.021 higher for every 1 kg more of GWG. For mothers who were underweight or a normal body weight before pregnancy, excessive GWG was positively associated with the overweightness/obesity of their offspring (OR (95% CI): 1.51 (1.21–1.90) and 1.30 (1.17–1.45)), while a reduced GWG was associated with an increased risk of their offspring being underweight (OR (95% CI): 1.24 (1.05–1.46) and 1.17 (1.04–1.32)) [51].

Our detailed analysis showed that, in the group of 7–11-year-old boys, statistically significant differences were found between boys with normal weight and boys with overweightness and obesity. Mothers of boys with excessive body weights gained, in pregnancy, an average of 5.5 kg more than the mothers of boys with a normal body weight. In the case of the entire age group of 7–11-year-olds, an analogous difference of 4.5 kg was demonstrated. The latter data were partially consistent with the results of the extensive meta-analysis by Voerman et al. The authors showed that a higher maternal pre-pregnancy BMI and gestational weight gain were associated with an increased risk of childhood overweight/obesity, with the strongest effects at later ages [52].

In addition, it was shown that the mothers of the boys examined had put on significantly more weight during pregnancy than the girls’ mothers (15.7 kg vs. 14.4 kg). It was also confirmed that if the mother gained more weight in pregnancy, today, her child is likely to have higher BMI percentiles compared to their peers. This correlation concerned the entire study group, the whole group of girls and the entire group of 7–11-year-olds (including, also, girls and boys separately).

Although there are many studies on this topic in the available literature, there are no data on the differences in the prevalence of overweightness and obesity in children in relation to maternal GWG taking into account the gender of the subjects, which, in a sense, was the subject of our research. Therefore, it is difficult for us to relate this specific data to the comparative data. This does not change the fact that numerous studies, including those cited in the discussion, confirm that abnormal, excessive maternal GWG promotes childhood obesity, which was also demonstrated in our own study.

In order to prevent obesity in children, future research should focus on a wider knowledge of modifiable risk factors, not only including those on the part of the mother, but they should also take into account the father and the child’s environment. There is also a great need to constantly educate future parents and build their awareness of the dangerous health effects that are associated with early childhood obesity.

The present study was able to document the influence of selected maternal factors influencing the incidence of overweightness and obesity of the participants. There are also a number of potential limitations in the study, which should be taken into account when interpreting the results. This study was geographically limited, carried out in one region of the country, and should be repeated in more regions. Another issue is the value of the mothers’ gestational weight gain given by the participants of the study. The authors were not in possession of data on the mothers’ BMI before pregnancy. In further studies, it would be advisable to determine the initial BMI before pregnancy in order to more accurately determine whether the mother increased her weight during pregnancy according to recommendations or not.

## 5. Conclusions

Excessive GWG shows long-term consequences in the form of childhood overweightness and obesity. Boys and children between 7–11 years of age are particularly vulnerable.

The conducted study did not confirm the role of the mother’s age in far-reaching health consequences for the child.

## Figures and Tables

**Table 1:** Characteristics of the mother during pregnancy in relation to the sex of the child.

Maternal Perinatal Risk Factors	Girls *n* = 358			Boys *n* = 391			*p*
	$\bar{x}$	Me	*s*	$\bar{x}$	Me	*s*	
Mother’s age at birth (y)	27.1	27	4.8	27.4	27	4.8	0.379
Gestational weight gain (kg)	14.4	13	5.8	15.7	15	7.1	0.021

*p*: test probability value calculated using the Mann-Whitney test; *n*: number; *x*: mean; Me: median; *s*: standard deviation; y: years of age.

**Table 2:** Differences in maternal age during pregnancy and gestational weight gain between children who currently have normal (or below) body mass and those who are overweight or obese. Body mass category based on the body mass index (BMI) percentile.

Children	Body Mass Category								
	Girls			Boys			All		
	NW UW	OW OB	*p*	NW UW	OW OB	*p*	NW UW	OW OB	*p*
**Mother’s age at birth (y)**									
all	27.2	26.4	0.245	27.3	28.2	0.436	27.2	27.3	0.754
4–6 y	26.7	26.6	0.894	27.2	29.8	0.199	26.9	28.7	0.275
7–11 y	27.5	26.3	0.293	27.8	27.6	0.732	27.6	27.0	0.330
12–15 y	27.2	26.5	0.583	26.4	27.2	0.772	26.8	26.7	0.885
**Gestational weight gain (kg)**									
all	14.2	15.7	0.134	15.3	17.6	0.163	14.8	16.6	0.057
4–6 y	14.6	15.8	0.379	15.9	14.3	0.468	15.2	14.8	0.959
7–11 y	13.4	16.5	0.063	15.1	20.6	0.005	14.3	18.8	0.002
12–15 y	15.1	15.0	0.685	15.4	15.8	0.980	15.3	15.3	0.797

kg: kilograms, NW: normal weight, OB: obesity, OW: overweight, *p*: test probability value calculated using the Mann-Whitney test, UW: underweight and y: years of age.

**Table 3:** Children’s BMI percentile values in relation to maternal factors, including age group division.

Children	BMI Percentile		
	Girls	Boys	All
**Mother’s age at birth (y)**			
All	0.01 (*p* = 0.902)	0.05 (*p* = 0.369)	0.03 (*p* = 0.455)
4–6 y	0.05 (*p* = 0.640)	0.09 (*p* = 0.390)	0.09 (*p* = 0.260)
7–11 y	−0.02 (*p* = 0.841)	−0.02 (*p* = 0.759)	−0.02 (*p* = 0.760)
12–15 y	0.01 (*p* = 0.877)	0.09 (*p* = 0.377)	0.06 (*p* = 0.356)
**Gestational weight gain (kg)**			
All	0.13 (*p* = 0.017)	0.09 (*p* = 0.068)	0.11 (*p* = 0.004)
4–6 y	0.12 (*p* = 0.249)	−0.01 (*p* = 0.944)	0.06 (*p* = 0.461)
7–11 y	0.19 (*p* = 0.017)	0.17 (*p* = 0.014)	0.18 (*p* < 0.001)
12–15 y	−0.02 (*p* = 0.802)	0.04 (*p* = 0.660)	0.01 (*p* = 0.831)

kg: kilograms; *p*: test probability value calculated using the Mann-Whitney test; y: years of age; BMI: body mass index.

**Table 4:** Children’s BMI percentile values depending on maternal factors, including age group division.

ANOVA Test	Mother’s Age at Birth (y)	Gestational Weight Gain (kg)
Sex	0.081	0.107
Age	0.563	0.210
body weight category by BMI percentile	0.787	0.040
sex × age	0.301	0.183
sex × body weight category by BMI percentile	0.099	0.986
age × body weight category by BMI percentile	0.521	0.008
sex × age × body weight category by BMI percentile	0.702	0.351

BMI: body mass index; y: years of age; kg: kilograms.
